# *Cinnamomum cassia* and *Syzygium aromaticum* Essential Oils Reduce the Colonization of *Salmonella* Typhimurium in an In Vivo Infection Model Using *Caenorhabditis elegans*

**DOI:** 10.3390/molecules26185598

**Published:** 2021-09-15

**Authors:** Marie Lang, Aude Montjarret, Emmanuel Duteil, Gilles Bedoux

**Affiliations:** 1 BioArmor S.A., Z.I de la Gare, F-22940 Plaintel, France; aamata@bioarmor.com (A.M.); eduteil@bioarmor.com (E.D.); 2 Laboratoire de Biotechnologie et Chimie Marines EA 3884, Université Bretagne Sud, F-56100 Lorient, France; gilles.bedoux@univ-ubs.fr

**Keywords:** essential oils, *Salmonella* Typhimurium, *Caenorhabditis elegans*, colonization assay, swimming motility, feed additives

## Abstract

The regulation of intestinal colonization in livestock by means of non-bactericidal additives is an important management lever for zoonotic bacteria such as *Salmonella* spp. *Caenorhabditis elegans* is proposed here as a model for the evaluation of five essential oils (EOs) as anti-colonization products against *Salmonella* Typhimurium. An evaluation of the toxicity of EOs for *C. elegans* showed LD_50_ values ranging from 74.5 ± 9.6 µg/mL for *Cinnamomum cassia* (CEO) to 271.6 ± 14.9 µg/mL for *Syzygium aromaticum* (SyEO). Both EOs significantly inhibited bacterial colonization in the digestive tract of *C. elegans* with reductions of 0.88 and 0.70 log CFU/nematode at nontoxic concentrations of 50 µg/mL and 150 µg/mL, respectively. With the minimal bactericidal concentrations of CEO and SyEO against *S.* Typhimurium being 312.5 µg/mL and 625 µg/mL, respectively, an antibacterial effect can be excluded to explain the inhibition of the bacterial load. The anti-colonizing activity of these two EOs could, however, be related to an inhibition of the swimming motility, which was significantly reduced by 23.47% for CEO at 50 µg/mL and 19.56% for SyEO at 150 µg/mL. This study shows the potential of *C. elegans* as a predictive in vivo model of anti-colonizing activities that is suitable for the evaluation of essential oils.

## 1. Introduction

*Salmonella enterica* serovar Typhimurium is an ubiquitous Gram-negative bacteria responsible for zoonoses [1,2]. The presence of *Salmonella* spp. in poultry, swine, cattle, and other animals involves significant economic losses, but also contamination of the food processing chains. Their ability to develop biofilm structures on many surfaces makes *Salmonella* spp. particularly persistent, and resistant to antibacterial agents [1,3]. The management of salmonellosis is important in the poultry industry, where the economic impacts are the greatest. Limiting the exposure of animals to the pathogen is a crucial step, and goes through an extensive disinfection of equipment and facilities [4]. Improving their protection by means of vaccines based on dead or attenuated bacteria is also helpful in terms of reducing microbial pressure. The use of pre/probiotics to modulate the microbiota in livestock was also developed to prevent bacterial colonization [4]. The search for anti-colonizing additives is, therefore, an interesting axis of research.

Besides their perfume and aroma, essential oils (EOs) develop different biological properties such as antioxidant [5,6], anti-inflammatory [6,7], antiviral [8,9], and antibacterial [10,11]. Activities of EO compounds against the foodborne pathogen *Salmonella* spp. have already been reported [1]. Carvacrol, cinnamaldehyde and thymol, alone or in combination, show antibacterial activities against *S.* Typhimurium [12] with minimal bactericidal concentrations of 200 and 400 µg/mL, respectively. Active concentrations were reduced to 100 µg/mL when cinnamaldehyde was combined to carvacrol or thymol [12]. Carvacrol was also successfully used against biofilm of *S.* Typhimurium grown on polystyrene and stainless steel. In these conditions, nonbiocidal concentrations of 60 to 120 µg/mL led to a 2 to 4-fold reduction in biofilm formation, according to a crystal violet assay [13]. This result showed the relevance of essential oils, alone or in combination, in the management of *Salmonella* spp. along the food production chain. In vitro measurements generally highlight the biological activities of plant extracts and allow a fast selection of suitable candidates. However, these tests are often mono-parametric or limited to simplified conditions, and biases are observed when switching to complex systems. Due to technical issues, significant costs and ethical reasons, the achievement of large-scale in vivo assays is not possible. The development of intermediate models, allowing a simplified monitoring of the host–pathogen interactions is, therefore, an important issue for the understanding of biological systems.

*Caenorhabditis elegans* is a small, free-living nematode, isolated from soil and feeding on bacteria. It is currently used as an experimental system for biological studies because of its simplicity, transparency, and ease of cultivation [14]. The nematode is susceptible to various pathogens involved in zoonoses and conserves many biological processes occurring in mammals [15,16,17]. Colonization of its digestive tract, cuticle or tail as well as other virulence mechanisms will then cause various symptoms, ranging from reduced life expectancy to rapid death [17,18,19]. These particularities make it an ideal model for the study of host–pathogen interactions [20]. Although, the pathosystem that uses *Pseudomonas aeruginosa* is today the most widely described [21,22,23], several studies show that *C. elegans* can also be an infection model for *Salmonella* Typhimurium [24]. After ingestion, the bacteria durably colonizes the digestive tract of *C. elegans* and induces an irreversible infection [24]. The persistent infection, linked to an oxidative burst in the digestive tissues, causes lethality within four to seven days [16,25]. Most recently, Desai et al. (2019) [26] showed that *Salmonella* forms sessile aggregates, comparable to in vivo biofilms, allowing chronical infections of the worm’s gut. Indeed, the pathogenicity of bacteria is down-regulated by an inhibition of the SPI-1-encoded tyrosine phosphatase (SptP), inducing an up-regulation of the host innate immunity through the p38-MAPK pathway [26]. The *C. elegans*/*S.* Typhimurium pathosystem was previously used to assess the bioactivity of red seaweeds [27]. Antibacterial seaweed water extracts (WE) incorporated in agar media impaired the ability of *S.* Enteritidis to colonize the digestive tract of the nematode and reduced its biofilm formation capacity and motility. The swimming motility in *S.* Typhimurium was recently shown to be related to more productive gut colonization [28]. Worm survival was also significantly improved 13 days after infection (by 65% for *S. gaudichaudii* WE at 800 µg/mL and by 45% for *C. crispus* at 800 µg/mL) and associated with an over-expression of immunity related genes [27]. An adaptation of the infection model in a liquid system is proposed here for the study of essential oils. 

To improve both food and health safety, and the evolution of regulation and antibiotics use, but also to meet consumers’ expectations, alternative solutions must be developed. Essential oils are promising ingredients to modulate the colonization of the digestive tract in the management of salmonellosis. Due to their poor solubility in agar media, a microplate-based liquid infection assay, using *C. elegans*, was developed to evaluate their activities. Colonization of the digestive tract was monitored by isolation and enumeration of the bacterial content. The effects of EOs on *S.* Typhimurium were then evaluated through antibacterial, antibiofilm and swimming motility inhibition assays.

## 2. Results and Discussion

### 2.1. EOs Composition

The chemical compositions of the essential oils (EOs) are presented in Table 1. *Cinnamomum cassia* essential oil (CEO) obtained from the leaves contains 79.98% cinnamaldehyde. *Origanum vulgare* (OEO) and *Satureja hortensis* (SaEO) essential oils have the same major compound, containing 70.72% and 47.37% carvacrol, respectively. *Thymus vulgaris* (TEO) and *Syzygium aromaticum* (SyEO) essential oils are also rich in phenolic compounds, with thymol (40.20%) and eugenol (80.67%), respectively, as the main compounds. The chemical profiles of the essential oils are in accordance with previous data [29,30,31,32].

### 2.2. Assessment of Toxic Concentrations

The toxicity of EOs was established using a liquid dilution assay on young adult worms. The LD_10,_ LD_50_ and LD_90_ values (doses involving 10%, 50% and 90% lethality, respectively), reported in Table 2, were deduced from the mortality curves established after 24 h of contact with the EOs solubilised with Simulsol^®^. While the use of the emulsifier alone did not induce any mortality of nematodes (data not shown), CEO was the most toxic one, with an LD_50_ of 74.5 ± 9.6 µg/mL. OEO and SaEO presented significantly similar activities whether for LD_50_ (113.9 ± 8.8 µg/mL and 136.9 ± 20.6 µg/mL, respectively) or for LD_90_ (263.4 ± 27.7 µg/mL and 265.7 ± 33.0 µg/mL, respectively). These similarities can be related to the equivalent chemical profiles between the two EOs. TEO showed an LD_50_ of 186.6 ± 33.2 µg/mL, and SyEO was less toxic, with an LD_50_ of 271.6 ± 14.9 µg/mL. There are currently no data regarding the toxicity of these EOs on the nematode *C. elegans* to which our results could be compared. However, the LD_50_ values obtained are in the same concentration ranges as observed with other EOs. Woods et al. (2013) [33] reported an LD_50_ of 457.0 µg/mL for the EOs of *Betula nigra*, which was rich in eugenol. In a study comparing the EOs of three *Cinnamomum* species, Satyal et al. (2013) [34] found out that *Cinnamomum glaucescens* EO developed high nematicidal activities, with an LD_50_ of 151.0 µg/mL. High toxicity was also observed with the EO of *Murraya paniculata* (LD_50_ = 37.0 µg/mL) by Dosoky et al. (2016) [35]. The LD_10_ established by the mathematical model allowed us to determine the maximal non-toxic doses for each essential oil, namely 50 µg/mL for CEO, OEO and SaEO, 100 µg/mL for TEO and 150 µg/mL for SyEO. These concentrations, at which no mortality was noted during the experiments, were used for the infection assay.

### 2.3. Effect of EOs on Bacterial Colonization

**Figure 1:**
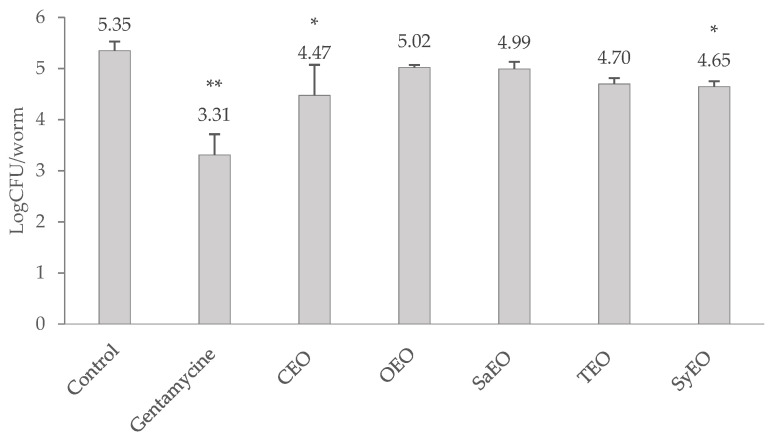
Evaluation of the bacterial load in the digestive tract of *Caenorhabditis elegans* infected with *Salmonella enterica* sv Typhimurium, after a 24 h treatment with essential oils. CEO, OEO and SaEO are tested at 50 µg/mL, TEO at 100 µg/mL, and SyEO at 150 µg/mL. Untreated nematodes are used as negative control, and gentamicin at 50 µg/mL is used as positive control. Values in LogCFU/nematode are means ± SD (*n* = 6). * *p* < 0.05, ** *p* < 0.01.

The load of *S.* Typhimurium in the gut of *C. elegans* was determined after a 24 h treatment with EOs and expressed as a logCFU/nematode (Figure 1). The effect of EOs was evaluated against gentamicin at 50 µg/mL, which was used as a reference antibiotic. Gentamicin induced a reduction in the bacterial load of two log points (5.35 ± 0.18 logCFU/nematode in control condition against 3.31 ± 0.41 logCFU/nematode with gentamicin 50 µg/mL). Only CEO (50 µg/mL) and SyEO (150 µg/mL) showed a significant decrease in the colonization of the digestive tract by *S.* Typhimurium, reducing the bacterial load to 4.47 ± 0.41 logCFU/nematode and 4.65 ± 0.10 logCFU/nematode, respectively. The anti-colonizing effect of these EOs can be linked to the major compounds found in both EOs, namely cinnamaldehyde and eugenol, which represent nearly 80% of the EOs. If the nematode model has been used previously to identify novel anti-infective agents [20], this is the first time that these EOs are evaluated for their effect against the gut colonization by *S.* Typhimurium. A *C. elegans*/*Pseudomonas aeruginosa* model already demonstrated the capacity of SyEO to prevent infection in vivo: at the sub-MIC concentration of ~16 mg/mL, EO enhanced the survival of the worm by 60% [36]. This study suggested that SyEO interferes with the virulence factors produced by PAO1 and induces cyanide asphyxiation and paralysis [36]. The same research team also reported that menthol (the main compound of peppermint EO) was able to modulate the infectivity and virulence of *Pseudomonas aeruginosa* PAO1 in the *C. elegans* model at a sub-MIC concentration of 800 µg/mL [37]. In vivo effects were then associated with an inhibition of the biofilm formation and the production of virulence factors by PA01 [37].

For that reason, it is necessary to study the regulation of *S.* Typhimurium intestinal colonization by EOs. The work was continued to find a potential mechanism of action of EOs, considering the order of activity observed for this essay: CEO > SyEO > TEO > SaEO > OEO. Indeed, EOs are known for their regulatory properties in relation to bacterial growth, biofilm development or bacterial motility [38]. These three parameters play an important role in the virulence and colonization of the digestive tract by *Salmonella* spp. [39].

### 2.4. Antimicrobial Activities of EOs

An adapted broth microdilution assay was used to determine the minimal inhibitory and bactericidal concentrations of EOs. The values presented in Table 2 show that the CEO, OEO, SaEO and TEO exhibit bactericidal activity from a concentration of 312.5 μg/mL. SyEO is less active against *S.* Typhimurium, with an MBC of 625.0 µg/mL. As the inhibitory concentrations were very close to the bactericidal concentrations, they could not be determined here. EOs are well known for their antibacterial activities, especially against *Salmonella* spp., and the activity of their major compounds has also been studied. The main mechanisms of action of carvacrol, eugenol, thymol and cinnamaldehyde were identified by Hyldgaard et al. (2012) [40]. Inhibition of metabolism and ATPase, interactions with lipids from the membrane and, therefore, membrane destabilization, ion losses and dissipation of the pH gradient, are recurring mechanisms [40]. The values obtained in our work are in the same order of magnitude as previous data. In their study, Si et al. (2006) [41] established an MBC of 300 µg/mL for SyEO and 100 µg/mL for CEO, against the strain of *S. typhimurium* DT104. These two plant extracts were also evaluated by Oussalah et al., (2007) [42], in addition to SaEO and TEO, against *S*. Typhimurium. They found MICs of 250 µg/mL for CEO, 500 µg/mL for SaEO and 1 mg/mL for TEO and SyEO. The differences in values can be attributed to the bacterial strain and to the protocol used, since, in the latter, the MICs were determined through an agar dilution assay [42]. It can be noticed that the bactericidal concentrations of the oils are higher than the toxic concentrations on the nematode (Table 2) and, therefore, the working concentrations used in the colonization assay. This allows us to exclude a bactericidal effect of CEO and SyEO in the regulation of the colonization of the digestive tract by *S.* Typhimurium.

### 2.5. Effects of EOs on Bacterial Biofilm Formation

**Figure 2:**
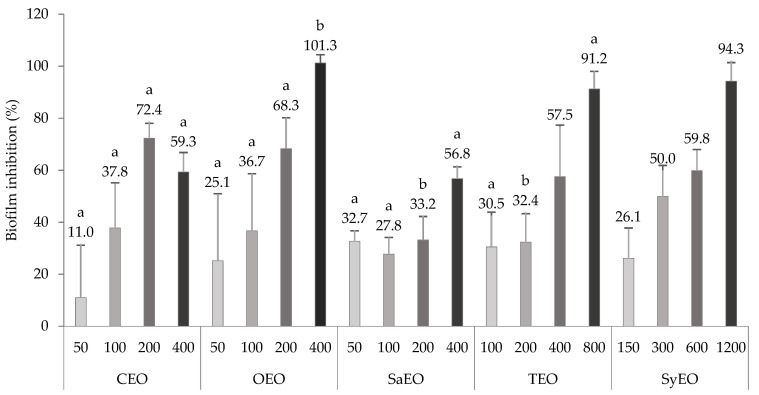
Evaluation of the antibiofilm activity of essential oils on the biofilm of *Salmonella* sv. Typhimurium formed in microplates. EOs are tested at concentrations greater than or equal to those applied to *Caenorhabditis elegans* during the colonization assay. Inhibition scores in percentage are means ± SD (*n* = 9). EOs at the same concentration with different letters are statistically different (*p* < 0.05).

*Salmonella* spp. are known for their ability to produce biofilm under biotic and abiotic conditions. Sessile aggregates were also observed along the gut of *C. elegans*, expressing specific genetic markers of biofilm [26]. Biofilm formation seems to be a survival strategy for *S.* Typhimurium, leading to a long-lasting colonization. Anti-biofilm activity could, therefore, be a way by which colonization is inhibited by EOs. To corroborate this mechanism, a liquid broth assay, which was associated with the quantification of biofilm by crystal violet, was realised for the different EOs. The concentrations tested on the worms during the colonization assay were used as minimal concentrations here in order to highlight some relations. The results are presented in Figure 2. All the tested EOs exhibited dose-dependent inhibitory activities on the biofilm of *S.* Typhimurium, even where non-bactericidal concentrations were applied (above 300 µg/mL for CEO, OEO, SaEO, and TEO or 600 µg/mL for SyEO) At the concentration of 200 µg/mL, CEO and OEO (72.4 ± 5.7% and 68.3 ± 11.9%, respectively) showed significantly higher inhibition rates than SaEO and TEO (33.2 ± 9.1% and 32.4 ± 10.9%, respectively). This result suggests a better antibiofilm activity of CEO and OEO at non-bactericidal concentrations. When bactericidal concentrations were reached (400 µg/mL), only OEO eradicated biofilm formation, with an inhibition of 101.3 ± 3.2%, suggesting a high correlation between antibacterial and antibiofilm effects. The antibiofilm activity of essential oils was previously established on *Salmonella* spp. since it is a recurrent pathogen in food processing. Specifically, the activity of TEO and OEO, as well as of the phenolic compound carvacrol, was established on the biofilm of *S.* Typhimurium by Soni et al. (2013) [13]. The monitoring of biomass using a crystal violet assay showed that both EOs significantly reduced the biofilm formation from a concentration of 120 µg/mL [13]. These results are consistent with our observations. At the threshold concentrations, the maximal effect was observed with SaEO (32.7 ± 4.0% inhibition at 50 µg/mL), while CEO showed a low and uneven activity (10.9 ± 20.2% at 50 µg/mL). EOs can be classified again for their antibiofilm activities at the minimal concentration: SaEO > TEO > SyEO > OEO > CEO. As the order of activity is not preserved when compared to the colonization assay (CEO > SyEO > TEO > SaEO > OEO), it seems that the effect of EOs on the bacterial load is not based solely on an antibiofilm activity, although this parameter cannot yet be completely excluded.

### 2.6. Effects of EOs on Swimming Motility

**Figure 3:**
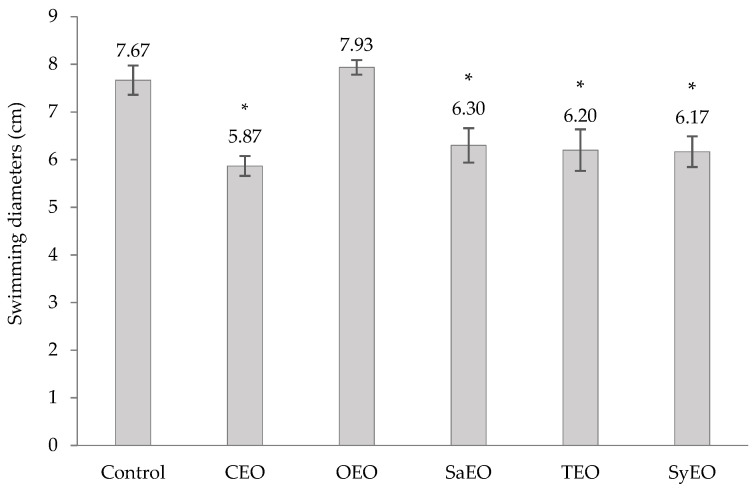
Effect of EOs on the swimming motility of *Salmonella* sv. Typhimurium. EOs are tested at concentrations equivalent to those applied to *Caenorhabditis elegans* during the colonization assay, i.e., 50 µg/mL for CEO, OEO and SaEO, 100 µg/mL for TEO, and 150 µg/mL for SyEO. Ethanol, applied at a final concentration of 1%, was used as control. Swimming diameters (cm) are means ± SD (*n* = 3). * *p* < 0.001.

Swimming motility can be observed on solid media when low concentrations of agar are used (<0.25%). Mediated by flagella, swimming motility initiates cell-to-surface contact [43] and was shown to be a major factor in the ability of *Salmonella* spp. to colonize digestive tracts [28,39]. Bacterial swimming was evaluated by measuring the diameters of migration after an incubation with EOs at the concentrations previously tested on the worm model (Figure S1). All of the EOs that were integrated into the growth medium, except OEO, significantly inhibited the motility of *S.* Typhimurium (Figure 3). The best effect was obtained with CEO (50 µg/mL; Ø 5.9 ± 0.2 cm), followed by SyEO (150 µg/mL; Ø 6.1 ± 0.3 cm), TEO (100 µg/mL; Ø 6.2 ± 0.4 cm) and SaEO (50 µg/mL; Ø 6.3 ± 0.4 cm). The conservation of the order of activity between motility and bacterial colonization assays in the worm model (CEO > SyEO > TEO > SaEO > OEO) suggests that the inhibition of swimming motility is a key factor in the regulation of gut colonization by the EOs tested in this study. To our knowledge, this is the first time that the effect of these five EOs on the motility of *S*. Typhimurium was established. However, cinnamaldehyde demonstrated activities on *E. coli*, which is also responsible of gut colonization [43]. When tested by Niu and Gilbert (2004) [43], cinnamaldehyde, at 286 ng/mL, reduced the swimming motility of *E. coli* ATCC 33456 by 60% ± 8% compared to controls. Another work showed a correlation between the inhibition of the cell invasion capacity of *Salmonella* Enteritidis, and the down-regulation of the genes flhC and motA, after a treatment with trans-cinnamaldehyde or eugenol [44]. As a transcriptional factor that leads to the activation of downstream motility genes, flhC also regulates the production of flagellin. The expression of the gene motA regulates flagellar assembly [44]. According to our results, we can suggest that sub-MIC concentrations of CEO and SyEO affect the expression of flhC and/or motA, leading to defective flagella and impairing the capacity of *S.* Typhimurium to invade the digestive tract of *C. elegans*.

## 3. Materials and Methods

### 3.1. Essential Oils

Five essential oils (EOs) obtained from BioArmor S.A. (Plaintel, France) were used in this study. *Cinnamomum cassia* (CEO), *Origanum vulgare* (OEO), *Satureja hortensis* (SaEO), *Thymus vulgaris* (TEO) and *Syzygium aromaticum* (SyEO) EOs were extracted by hydrodistillation. Table 1 lists the major components (concentrations ≥ 2%) of EOs obtained by gas chromatography, performed by the supplier. Simulsol^®^ (Seppic, Courbevoie, France) an ethoxylated castor oil-based emulsifier, was used to disperse EOs in the different media. Stock solutions were prepared freshly, by mixing equivalent amounts of EOs and Simulsol^®^, in the appropriate media. The emulsions were prepared by stirring combined withtwo successive ultrasonic treatments of 4 min. The negative control consisted of diluted Simulsol^®^.

### 3.2. Bacterial Strains and Maintenance 

*Escherichia coli* strain OP50 (*Caenorhabditis* Genetics Center (CGC)) and *Salmonella enterica* serovar Typhimurium were used in the different assays. *S.* Typhimurium was isolated from a rag taken from a pig farm and was kindly provided by Labocea (Ploufragan, France). Strains were grown overnight in Luria–Bertani (LB) broth at 37 °C, under orbital agitation (110 rpm).

### 3.3. Caenorhabditis Elegans Strain and Maintenance

*Caenorhabditis elegans* strain SS104 glp-4(bn2ts) was provided by the CGC, which is funded by the NIH Office of Research Infrastructure Programs (P40 OD010440). This temperature sensitive mutant was used to have a constant number of worms along the colonization assay as it produces progeny at 15 °C but not at 25 °C. Maintenance and synchronisation methods were adapted from Porta-de-la-Riva et al. (2012) [45].

Nematodes were maintained at 16 °C on Nematode Growth Medium (NGM) seeded with *E. coli* OP50. Synchronisation was realised when enough gravid nematodes or eggs were present on the plates. Eggs were recovered by bleaching. The bleaching solution consisted of water, 5% hypochlorite sodium solution in water and 5 M KOH buffer in 2:2:1 proportion, according to conventional procedures [45]. The purified eggs were then incubated at 16 °C under agitation in sterile M9 buffer, until hatching. The L1 larvae thus obtained were rinsed and placed on a layer of *E. coli* OP50, then incubated at 25 °C until the young adult (YA) stage was reached (3–4 days).

### 3.4. Evaluation of Toxic Concentrations

The lethal doses (LD) of each essential oil were determined prior to the colonization assay, using a liquid media dilution assay. Approximately 20 Young Adult (YA) nematodes were exposed to serial dilutions of essential oil in S-Basal media. After a 24-h exposure at 25 °C, the mortality rate was determined by counting, using optical microscopy. Nematodes were considered as dead in the absence of movement and pharyngeal pumping. LD_10_, LD_50_ and LD_90_ values, corresponding to the doses involving 10%, 50% and 90% death rates, respectively, were determined using the dose effect tool in XLSTAT 2020. Six independent replicates were performed (*n* = 6). This step was intended to determine the working concentrations of the different EOs by selecting the maximum concentrations that induced toxicity of less than or equal to 10%. These concentrations are 50 µg/mL for CEO, OEO and SaEO, 100 µg/mL for TEO and 150 µg/mL for SyEO.

### 3.5. Colonization Assay

The screening of anti-infective activities against *S.* Typhimurium was performed in liquid medium in 96-well plates. A description of the complete procedure is given in Supplementary Figure S2. For the infection step, YA nematodes, previously washed thrice in M9 buffer to remove OP50, were spotted on a lawn of *S.* Typhimurium grown on Slow Killing (SK) agar plates for 6h. The infected nematodes were then recovered and washed thrice with M9 buffer and about 10 worms/well were subjected to the different conditions. EOs, as well as gentamicin at 50 μg/mL, were diluted in Liquid Kill (LK) media (2/3 SK broth and 1/3 S-Basal buffer) and used for the treatment. LK media with Simulsol^®^ was used as control. Culture media and buffers were prepared as described by Conery et al. (2014) [46].

The enumeration of bacterial Colony Forming Units (CFU) within the gut of *C. elegans* was performed according to the method presented by Konga et al. (2014) [47] with some modifications. After a 24-h exposure to EOs or antibiotic in liquid medium, approximately 10 living worms were briefly anesthetized in 25 mM Levamisole (Lev). The worms were washed at least thrice in 200 μL antibiotic cocktail comprising 25 mM Lev and gentamicin at 10 μg/mL for an overall time of 45 min (15 min/ step) to completely kill the bacterial cells that were associated with the worm cuticle. Then, the worms were washed thrice with 200 μL of 25 mM Lev to eliminate the killed bacteria and residual antibiotic. The numbers of worms were recorded before mechanical disruption in 50 μL of 1% Triton X (X100; Sigma-Aldrich, Saint-Louis, MO, USA), using a pestle. Serial dilutions of the worm lysates were performed and 100 μL of each dilution were spotted on LB agar. Colonies were counted after an overnight incubation of the plates at 37 °C. The bacterial CFU per worm was calculated using the formula reported by Ooi et al. (2012) [19]. Six independent replicates were performed (*n* = 6).

### 3.6. MIC and MBC Determination

The minimum inhibitory (MIC) and bactericidal (MBC) concentrations were determined for each EO on *Salmonella* Typhimurium using a broth microdilution assay. The emulsion of essential oils in water-based media implied the appearance of a cloudiness; MTT was used to reveal the presence of metabolically active bacteria. EOs were diluted in LB medium within a range of 5 to 0.05 mg/mL. A volume of 195 µL of each dilution was placed in the wells of a flat bottomed 96-well microplate and bacterial suspension was added to obtain a final Optical Density (OD) of 0.0125 at 600 nm (Shimadzu UV-1800 spectrophotometer Shimadzu Europa GmBh, Duisburg, Germany). Microplates were incubated for 24 h at 37 °C without agitation. After cell growth, a tetrazolium salt (MTT, Sigma- Aldrich, Saint-Louis, MO, USA) solution in DMSO (Fisher Scientific, Hampton, NY, USA) was added to each well at a final concentration of 90 µg/mL and plates were incubated for 30 min. This allowed viable microorganisms to metabolize the yellow MTT into purple formazan crystals. An aliquot of each well that did not present a formazan production was then spread on LB agar plates and incubated for 24 h at 37 °C for the determination of MIC or MBC. The MIC and MBC corresponded to the lower concentrations that led to growth or the absence of growth, respectively, after spreading. Six independent replicates were performed (*n* = 6).

### 3.7. Inhibition of Biofilm Formation 

The antibiofilm properties of essential oils on *Salmonella* Typhimurium were evaluated using a microtiter plate assay adapted from Kalai Chelvam et al. (2014) [39]. Briefly, an overnight culture of *S*. Typhimurium was subcultured in LB to a concentration of 0.1 OD_600nm_ and 100 µL of this solution was inoculated in the wells of a microtiter plate. Quantities of 100 µL of the EOs’ solutions were then added to reach final concentration ranging from 50 to 400 µg/mL for CEO, OEO and SaEO, from 100 to 800 µg/mL for TEO, and from 150 to 1200 µg/mL for SyEO. To evaluate the potential activity of Simulsol^®^ on biofilm formation, dilutions of the emulsifier in the adequate range of concentrations (50 to 400 µg/mL, 100 to 800 µg/mL, or 150 to 1200 µg/mL) were used as blanks. Wells with LB alone or LB inoculated with *S.* Typhimurium were used as positive and negative controls, respectively. 

After a 48 h period of incubation at 37 °C, unbound cells were removed by inversion of microtiter plate, followed by vigorous tapping on absorbent paper. Subsequently, adhered cells were heat fixed in an oven for 1h at 80 °C. Adhered cells were stained by addition of 200 μL of crystal violet (0.5% in water) for 15 min. The stain was removed by thorough washing with distilled water. To quantify the adhered cells, 200 μL of decolouring solution (ethanol/acetone, 80:20%) was added to each well for 15 min. The absorption of the released stain was measured at 600 nm wavelength. Based on the OD at 600 nm, inhibition (in%) was calculated from the absorbance. Nine independent replicates were performed (*n* = 9).

### 3.8. Swimming Motility Assay

To evaluate the effect of EOs on bacterial motility, a swimming assay was carried out on agar plate, as previously described [39]. Swim plates were prepared freshly, with LB supplemented with 0.5% glucose and 0.25% agar. Media was complemented after sterilisation with EOs at final concentrations of 50 µg/mL (CEO, OEO and SaEO), 100 µg/mL (TEO) or 150 µg/mL (SyEO) that were previously solubilised in absolute ethanol (final concentration of 1%). Swim media complemented with ethanol at 1% was used as negative control. Swim plates were dried for 2 h under laminar flow before inoculation with *S*. Typhimurium that was previously diluted to a concentration of 0.1 OD at 600 nm. Plates were then incubated for 14 h at 30 °C in a humid atmosphere. Swimming diameters were measured (in cm) and images were taken using a GeneFlash system (Syngene, India). Three independent replicates were performed (*n* = 3).

### 3.9. Statistical Analysis

Conventional statistical methods were used to calculate means and standard deviations. For the colonization and antibiofilm assays and for the evaluation of swimming motility, an analysis of variance (ANOVA) was performed to determine differences (*p* < 0.05), and means were compared to the control condition using Dunnett’s test in combination with Tukey’s test. The statistical analyses were realised using XLSTAT 2020 for Windows.

## 4. Conclusions

This work contributes to the development of an in vivo model that is suitable for the study of essential oils’ effects on the gut colonization by *Salmonella* Typhimurium. The results showed that the EOs of *Cinnamomum cassia* and *Syzygium aromaticum*, at non-toxic and non-bactericidal doses, regulate the development of *S*. Typhimurium in the digestive tract of *C. elegans.* This result should allow the development of solutions intended to reduce the occurrence of *Salmonella* spp. in production animals, thereby avoiding contamination of the food chain. While EOs limit both the biofilm formation and motility of *S.* Typhimurium, the observed anti-colonizing effect was mainly related to an inhibition of swimming motility. A defect in swimming motility, regulated by the movement of flagella, is linked to lower adhesion capacity in epithelial cells [28]. According to the literature, the most likely mechanism of action for CEO and SyEO remains an inhibition of the expression of the motility-specific genes flhC and motA [44]. This proposition must, however, be confirmed by more in-depth studies. Previous publications showed that *S.* typhimurium is able to regulate the immune system of *C. elegans* to promote its infection [26], and that its installation in the digestive system is combined with oxidative stress [25]. As both immunomodulative [48] and antioxidant activities of EOs are recognized, further work should also focus on the development of a multiparametric assay that integrates the measurement of oxidative bursts related to infection in *C. elegans*.

## Figures and Tables

**Table 1:** Chemical compositions of commercial essential oils (%) determined by gas chromatography. The compounds are classified according to their concentration in all the tested EOs. These data are provided by the supplier.

	*Cinnamomum cassia*	*Origanum vulgare*	*Satureja* *hortensis*	*Thymus* *vulgaris*	*Syzygium* *aromaticum*
	(CEO)	(OEO)	(SaEO)	(TEO)	(SyEO)
Eugenol		0.02			80.67
Cinnamaldehyde (cis + trans)	79.98				
Carvacrol		70.72	47.37	2.13	
Thymol		2.12	1.61	40.20	
p-Cymene		3.70	11.59	22.04	
γ-Terpinene		4.97	27.36	11.46	
β-Caryophyllene		3.58	1.58	3.55	9.6
o-Methoxycinnamaldehyde	8.00				
Eugenyl acetate					6.0
Linalool		3.00	1.04	3.66	
Cinnamyl acetate	2.52				
Myrcene			2.00	2.14	
α-Terpinene		0.58	2.09	1.80	
Coumarin	1.56				
D-Limonene	0.04	0.72		0.48	
Eucalyptol		1.41			
(D,L)-Borneol		1.20		1.34	
α-Pinene				1.18	
β-Pinene		1.15			
4-Terpinenol				1.08	
Benzaldehyde	0.83				
Camphor		0.83			
α-Terpineol		0.76		0.17	
4-Carvomenthenol		0.75			
Camphene				0.70	
Phenylethyl alcohol	0.70				
trans-Sabinene hydrate				0.50	
Styrene	0.21				
Salicylaldehyde	0.20				
Terpinolene				0.15	
Cinnamic acid	0.11				
Cinnamyl alcohol	0.11				
Benzyl Benzoate	0.06				
Furfural					0.06

**Table 2:** Evaluation of the toxicity of essential oils on *Caenorhabditis elegans* and the pathogen *Salmonella enterica* sv. Typhimurium. Values in µg/mL are means ± SD (*n* = 6). Values with different letters in the same line are significantly different (*p* < 0.05).

		CEO	OEO	SaEO	TEO	SyEO
*C. elegans*	LD_10_	43.7 ± 5.4 ^a^	52.8 ± 4.2 ^ab^	71.6 ± 18.4 ^bc^	91.8 ± 1.1 ^c^	156.8 ± 16.0 ^d^
	LD_50_	74.5 ± 9.6 ^a^	113.9 ± 8.8 ^b^	136.9 ± 20.6 ^bc^	186.6 ± 33.2 ^c^	271.6 ± 14.9 ^d^
	LD_90_	127.3 ± 18.2 ^a^	263.4 ± 27.7 ^b^	265.7 ± 33.0 ^b^	305.7 ± 65.8 ^b^	471.2 ± 3.3 ^c^
*S.* Typhimurium	MIC MBC	- 312.5 ± 0.0	- 312.5 ± 0.0	- 312.5 ± 0.0	- 312.5 ± 0.0	- 625.0 ± 0.0

## Supplementary Materials

The following are available online. Figure S1: Pictures of swimming motility plates inoculated with *Salmonella* sv. Typhimurium; Figure S2: Workflow of in vivo colonization assay.
