# Supplementary material for: Cinnamomum cassia and Syzygium aromaticum Essential Oils Reduce the Colonization of Salmonella Typhimurium in an In Vivo Infection Model Using Caenorhabditis elegans
Source: Molecules. 2021 Sep 15;26(18):5598. doi: 10.3390/molecules26185598 (PMC8467367; doi:10.3390/molecules26185598)
Supplement: Supplementary file 1 [file molecules-26-05598-s001.zip › molecules-1363447-supplementary.pdf]

molecules

MDPI

Supplementary Materials

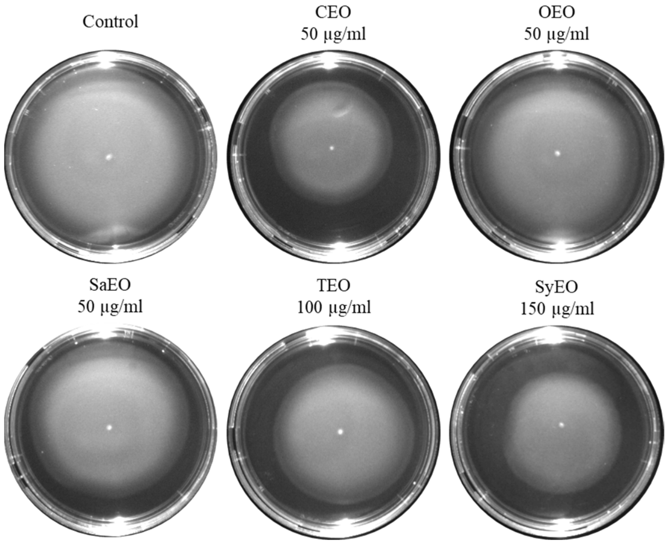

Figure S1. Pictures of swimming motility plates inoculated with *Salmonella* sv. Typhimurium. EOs are tested at concentrations equivalent to those applied to *Caenorhabditis elegans* during the colonization assay. Images are taken using a GeneFlash system after 14 h of incubation at 30 °C.

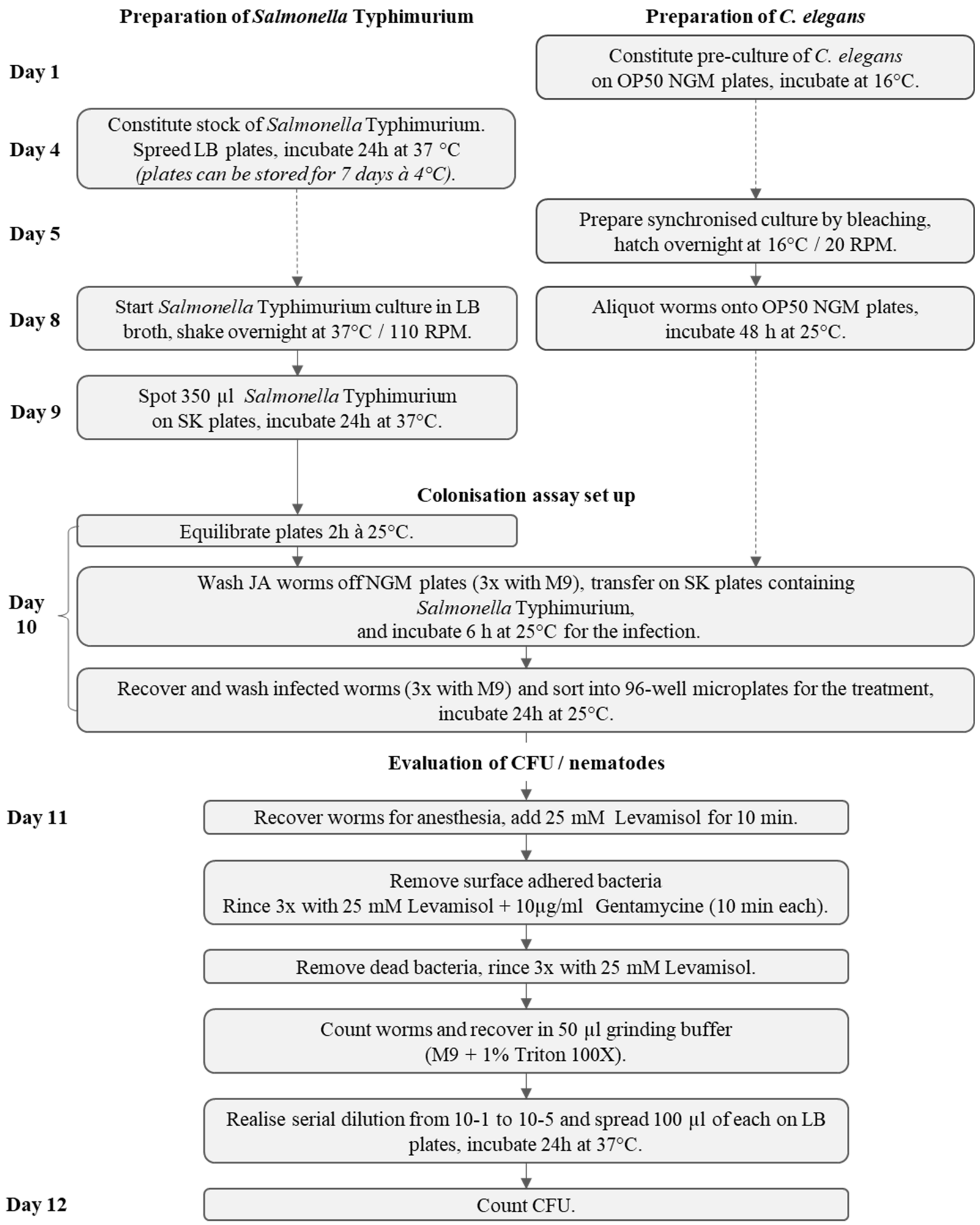

Figure S2. Workflow of the *in vivo* colonization assay. The methodology used for the infection assay with *Salmonella* Typhimurium is diagrammed, to represent all the successive steps undergone by bacteria and worms, from cultivation.
